# Do Second-Generation Antipsychotics Protect Against the Emergence of (Hypo)mania in Depressed Patients with Mixed Features?

**DOI:** 10.1192/j.eurpsy.2025.442

**Published:** 2025-08-26

**Authors:** M. Zimmerman

**Affiliations:** Brown University, ProvidencePPPPro, United States

## Abstract

**Introduction:**

Five medications have been approved for the treatment of bipolar depression, though no medication has been approved for the treatment of depression with mixed features. Post-hoc analyses of the bipolar depression trials examined efficacy in depressed patients with and without mixed features. Each study also examined the emergence of manic symptoms as a possible negative outcome, and each found that the frequency of emergent manic symptoms was more frequent in the patients treated with placebo than with medication though in no study was the difference statistically significant. However, the studies were not powered to detect a significant difference in treatment emergent (hypo)manic episodes thereby prompting the current pooled analysis.

**Objectives:**

The goal of the present pooled analysis was to examine whether second-generation antipsychotics that have been found effective in treating depression with mixed features protect against the emergence of (hypo)manic episodes in depressed patients with concurrent manic symptoms.

**Methods:**

Five placebo-controlled studies of the effectiveness of second-generation antipsychotics in the treatment of depressed patients with mixed features reported information on the emergence of manic symptoms.

**Results:**

The 5 studies included 1,829 depressed patients with mixed features—1,620 with bipolar disorder and 209 with major depressive disorder. In each study, the frequency of treatment-emergent manic episodes was higher in the group treated with placebo, though in no study was this difference significant. Summed across studies, the frequency of treatment emergent (hypo)manic episodes was higher in the patients receiving placebo (4.0% vs. 2.4%, X^2^=3.66, p=.056). Excluding the patients treated with olanzapine, which has not been found to be effective in treating bipolar depression, the frequency of emergent (hypo)manic episodes was significantly higher in the patients receiving placebo, (4.0% vs. 1.8%, *X* ^2^=7.31, p=.007).

**Conclusions:**

The results of the present analysis suggest that the second-generation antipsychotics that are effective in treating bipolar depression also protect against a (hypo)manic switch in depressed patients with mixed features.

**Disclosure of Interest:**

None Declared

